# The Impacts of Swimming Exercise on Hippocampal Expression of Neurotrophic Factors in Rats Exposed to Chronic Unpredictable Mild Stress

**DOI:** 10.1155/2014/729827

**Published:** 2014-11-11

**Authors:** Pei Jiang, Rui-Li Dang, Huan-De Li, Li-Hong Zhang, Wen-Ye Zhu, Ying Xue, Mi-Mi Tang

**Affiliations:** Institute of Clinical Pharmacy & Pharmacology, Second Xiangya Hospital, Central South University, Changsha 410011, China

## Abstract

Depression is associated with stress-induced neural atrophy in limbic brain regions, whereas exercise has antidepressant effects as well as increasing hippocampal synaptic plasticity by strengthening neurogenesis, metabolism, and vascular function. A key mechanism mediating these broad benefits of exercise on the brain is induction of neurotrophic factors, which instruct downstream structural and functional changes. To systematically evaluate the potential neurotrophic factors that were involved in the antidepressive effects of exercise, in this study, we assessed the effects of swimming exercise on hippocampal mRNA expression of several classes of the growth factors (BDNF, GDNF, NGF, NT-3, FGF2, VEGF, and IGF-1) and peptides (VGF and NPY) in rats exposed to chronic unpredictable mild stress (CUMS). Our study demonstrated that the swimming training paradigm significantly induced the expression of BDNF and BDNF-regulated peptides (VGF and NPY) and restored their stress-induced downregulation. Additionally, the exercise protocol also increased the antiapoptotic Bcl-xl expression and normalized the CUMS mediated induction of proapoptotic Bax mRNA level. Overall, our data suggest that swimming exercise has antidepressant effects, increasing the resistance to the neural damage caused by CUMS, and both BDNF and its downstream neurotrophic peptides may exert a major function in the exercise related adaptive processes to CUMS.

## 1. Introduction

Depression is a debilitating and widely distributed disorder which is associated with exposure to stressful life events. Studies of chronic stress in animal models and postmortem tissues from depressed patients demonstrated that reduced size of limbic brain regions that regulate mood and cognition and decreased neuronal synapses in these brain areas may contribute to the pathogenesis of depression [1]. There is emerging evidence that exercise has antidepressant effects, whereby promoting neurogenesis and inhibiting neurodegeneration [2]. Although exercise seems to have therapeutic and preventive effects on the course of depression, the underlying mechanisms remain elusive. It has been proposed that the key mechanism mediating the broad benefits of exercise on the brain is induction of neurotrophic factors, which instruct downstream structural and functional changes [3]. The protective effects of exercise from chronic stress have been best-studied in the hippocampus, where exercise increased synaptic plasticity and neurotrophic factors expression. Previous studies indicate that exercise can promote hippocampal neurotrophic cascades and enhance neural survival, differentiation, connectivity, and plasticity, while stress shows the opposite effects, which indicates a potential mechanism for exercise to alleviate stress [4].

Brain-derived neurotrophic factor (BDNF) is the most abundantly expressed neurotrophin in the mature central nervous system and supports the survival of many types of neurons. A number of animal studies have documented that the exposure to chronic stress can result in decreased BDNF expression in hippocampus [5]. Conversely, both antidepressant treatment and exercise can enhance hippocampal BDNF status [6, 7]. The neuropeptide VGF and neuropeptide Y (NPY) have been implicated in the actions of BDNF and both of which can be induced by BDNF and antidepressants [8, 9]. Accumulating evidence has shown that BDNF was implicated in the pathophysiology of depression and the antidepressant action of exercise. Nevertheless, the role of its downstream neuropeptides and other neurotrophic factors remains unclear. Other neurotrophins, including nerve growth factor (NGF), glia cell-derived neurotrophic factor (GDNF), and neurotrophin-3 (NT-3), are also important factors for regulation of neuroplasty and were implied to play a role in the neurotrophic hypothesis of depression [10]. There are several additional growth factors that also have been implicated in neurogenesis, depression, and treatment response, such as insulin-like growth factor-1 (IGF-1), vascular endothelial growth factor (VEGF), and fibroblast growth factor-2 (FGF-2) [11].

It was reported that swimming exercise could reverse the chronic unpredictable mild stress (CUMS) induced depression-like state in rodents [12–14]. However, the results are inconsistent and the underlying mechanisms are far from fully understood [15]. Since neurotrophic factors are suggested to exert a major function in the antidepressant effects of exercise, the main objective of the present study was to further establish the therapeutic role of swimming exercise in depression and systematically evaluate the potential neurotrophic factors that were involved in the antidepressive effects of the exercise paradigm. The expression of biomarkers of cell survival including the antiapoptotic protein Bcl-xl and the proapoptotic protein Bax was also assessed [16].

## 2. Materials and Methods

### 2.1. Animals

Experiments were carried out with male Sprague-Dawley rats (250–280 g), supplied by the Experimental Animal Center of the Second Xiangya Hospital. The rats were housed at 22–25°C and humidity 50–60% with a 12 h light-dark cycle and had free access to commercial rat chow and water, except when they were submitted to CUMS. All animal use procedures were carried out in accordance with the Regulations of Experimental Animal Administration issued by the State Committee of Science and Technology of the People's Republic of China, with the approval of the Ethics Committee in our university.

### 2.2. CUMS Procedure and Exercise Protocol

The rats were randomly divided into four groups (*n* = 8): Control group, Exercised group, Stressed group, and Stressed + Exercised group. While the rats in Control group were undisturbed, the Exercised group was trained in a progressively increasing moderate swimming protocol as previously reported with minor change [13]. Swimming exercise was performed in a plastic water tank (100 cm × 80 cm × 90 cm) at 32 ± 1°C and a depth of 55 cm. The protocol included two phases: adaptation and training. In the adaptive phase, the rats swam 20 min per day for 6 days. The adaptation was aimed at reducing the water induced stress without promoting physiological alterations in relation to the physical training [17]. After rest for one day, the training period began from the first day of the second week. Swimming duration was progressively increased from 20 min to 70 min per day for 6 days in 1 week. This intensity was maintained to the end of the training program. The swimming program lasted 6 days per week for a total of 4 weeks. The beneficial effects of the swimming protocol have been repeatedly validated by previous reports [13, 18].

The Stressed group received a previously established weekly stress regime with minor modification [19]. Briefly, the CUMS rats were randomly exposed to one of these stressors per day for 4 weeks and the same stressor was not used in 2 consecutive days: cage tilting (45°) for 24 h; water deprivation for 24 h, finally with 1 h an empty bottle; 1 min tail clamping; physical restraint for 2 hours; fasting for 24 h; soiled cage for 24 h; and 20 min noise. The Stress + Exercised rats received both CUMS and the exercise protocol. As isolation may cause additional stress to the animals [20], the Stressed and Stressed + Exercised group were housed separately (cage size: 26 × 19 × 15 cm), while four rats in the other two groups shared one cage (cage size: 90 × 45 × 25 cm).

### 2.3. Sucrose Preference Test (SPT)

SPT is a measure of stressed-induced anhedonia state, a key depressive-like behavior in rats [19]. Prior to SPT, all the rats were housed individually and habituated to 48 h of forced 1% sucrose solution consumption in two bottles on each side. Then after 16 h water deprivation, we placed two preweighted bottles, one containing 1% sucrose solution and another containing tap water, to each rat. The side (left and right) of the two bottles was randomly placed, in order to avoid spatial bias. The bottles were weighted again after 1 h and the weight difference was considered to be the rat intake from each bottle. The preference for sucrose was measured as a percentage of the consumed 1% sucrose solution relative to the total amount of liquid intake.

### 2.4. Open Field Test (OFT)

OFT is a measure to evaluate the general locomotion and exploratory behavior of rats [12]. As previously described, the test was conducted between 8:00 and 12:00, one day after SPT. Each rat was placed at the centre of an apparatus with a square arena (90 cm × 90 cm × 40 cm). The floor of the arena was equally divided into 25 squares. The locomotor activity was video-taped and the time in the centre square and number of crossings (squares of crossings with all paws) were manually counted for 5 min. Between the tests, the apparatus was thoroughly cleaned and dried.

### 2.5. Real-Time PCR Analysis

Total RNA from hippocampus was isolated using Trizol reagent (Invitrogen, USA) according to the manufacturer's instructions. Quantification of mRNAs was performed on Bio-rad Cx96 Detection System (Bio-rad, USA) using SYBR green PCR kit (Applied Biosystems, USA) and gene-specific primers. Each cDNA was tested in triplicate with 40 cycles of amplification. Relative quantitation for PCR product was normalized to *β*-actin as internal standard. The sequences of gene-specific primers are summarized in Table 1.

### 2.6. Statistics

Statistical analysis was performed with SPSS 13.0 software. All values were presented as mean ± SEM. Body weight gain was analyzed using a two-way repeated ANOVA analysis with Stress and Exercise as between subject factors and time as within subject factor. The data of SPT, OFT, and relative mRNA expression were analyzed by using two-way ANOVA followed by Fisher's least significant difference (LSD) post hoc test. The prior level of significance was established at *P* < 0.05.

## 3. Results

### 3.1. Behavioral Test and Body Weight Gain

**Figure 1:**
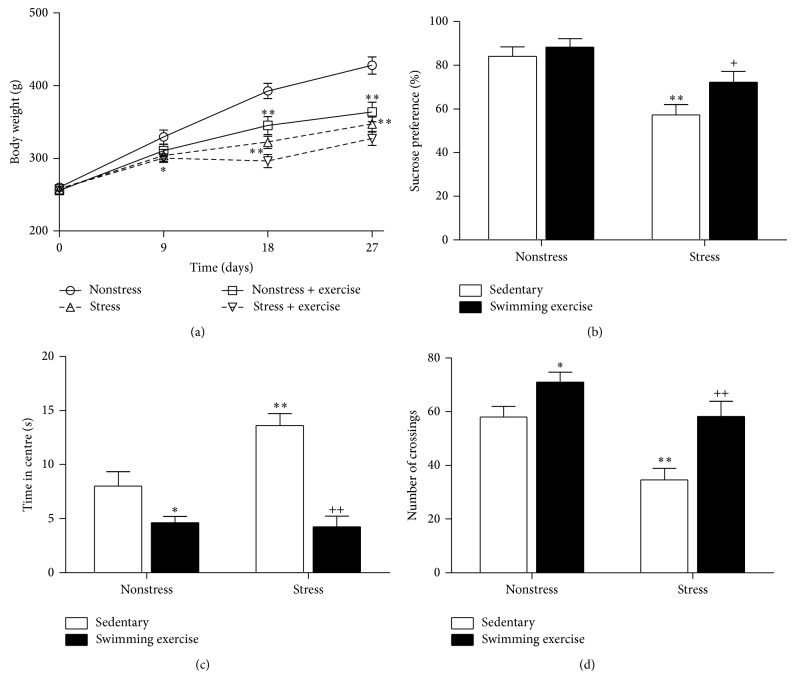
Effect of swimming exercise and CUMS on rat body weight (a) and behavioral changes in sucrose preference test (b) and time spent in centre (c) and number of crossings (d) in open field test. Data are means ± SEM (*n* = 8). ^*^ *P* < 0.05, ^**^ *P* < 0.01 compared to Control group. ^+^ *P* < 0.05, ^++^ *P* < 0.01 compared to Stressed group.

Both exercise and CUMS procedure significantly slowed down the body weight growing rate (Figure 1(a)). Stress exposure induced a significant decrease in sucrose preference. Exercise had no effect on sucrose intake in nonstressed rats, but significantly reversed the decline in the sucrose preference of CUMS rats (Figure 1(b)). The locomotor activity and exploratory behavior were observed in the OFT (Figures 1(c) and 1(d)). 4 weeks of CUMS led to a significantly decreased number of crossings and increased time spent in centre. The Exercised group seems to be more explorative, with less time spent in centre and more numbers of crossings. The Stressed + Exercised group also showed improved exploratory behavior compared with Stressed group. Overall, the results suggest that swimming exercise can successfully ameliorate the stress-induced depression-like state in rats.

### 3.2. Neurotrophic Effects of Swimming Exercise

**Figure 2:**
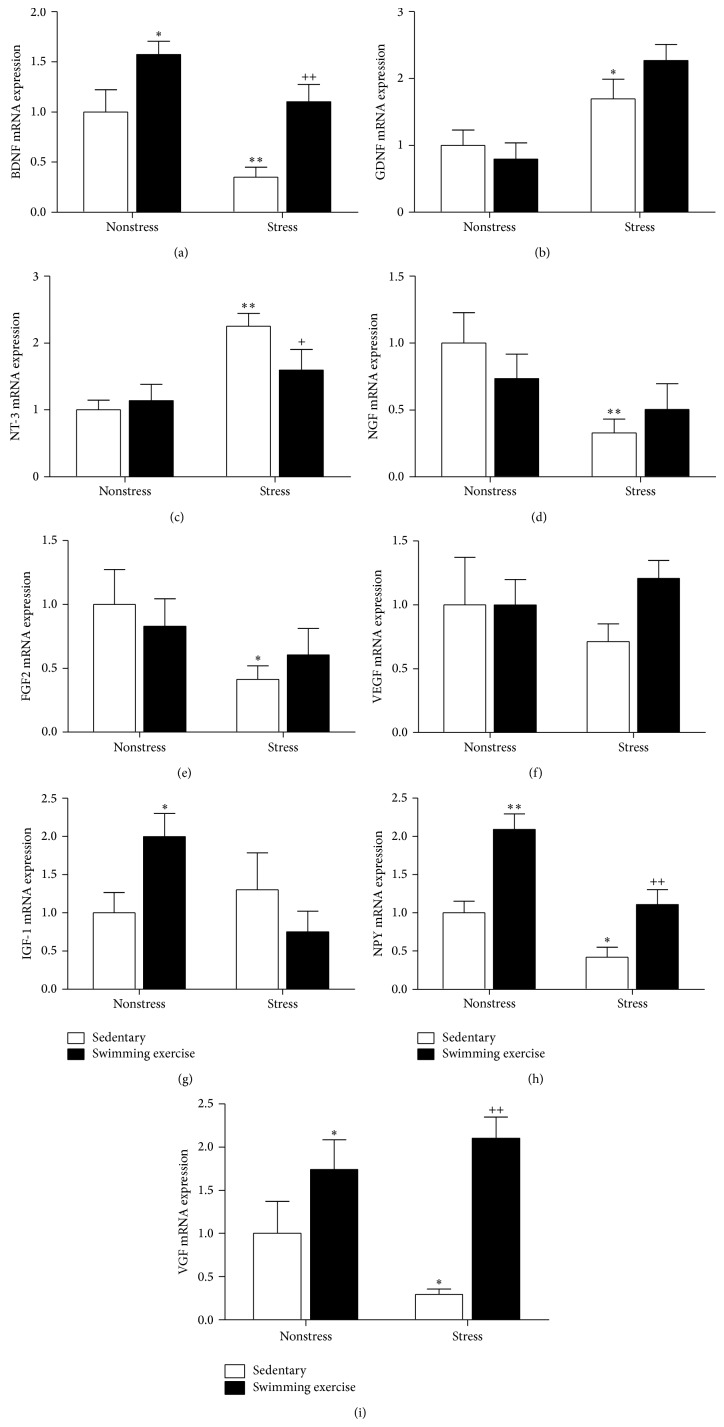
Effect of swimming exercise and CUMS on the neurotrophic factors expression. Data are means ± SEM (*n* = 6). ^*^ *P* < 0.05, ^**^ *P* < 0.01 compared to Control group. ^+^ *P* < 0.05, ^++^ *P* < 0.01 compared to Stressed group.

**Figure 3:**
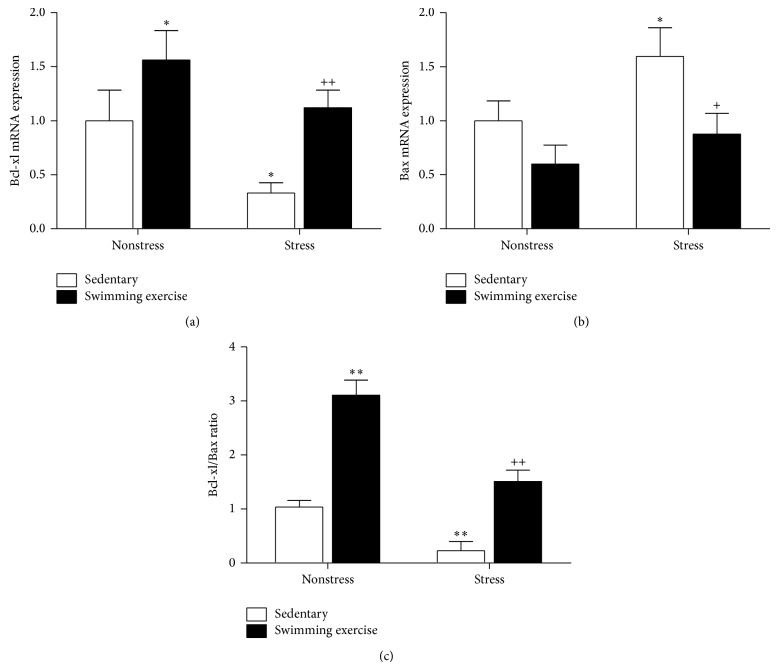
Effect of swimming exercise and CUMS on the expression of Bcl-xl (a) and Bax (b) and ratio of Bcl-xl/Bax (c). Data are means ± SEM (*n* = 6). ^*^ *P* < 0.05, ^**^ *P* < 0.01 compared to Control group. ^+^ *P* < 0.05, ^++^ *P* < 0.01 compared to Stressed group.

Since neurotrophic factors play a major role in the antidepressant action of exercise, we assessed the expression of the neurotrophic factors that related to depression in the hippocampus. As previously reported [21], exercise significantly induced the expression of BDNF, the mostly documented biomarker in depression, and reversed the inhibitory effects of chronic stress (Figure 2(a)). Interestingly, stress induced GDNF (Figure 2(b)) and NT-3 (Figure 2(c)) expression, while no influence of exercise on the two neurotrophins was found, which suggests a potential compensatory adaptative mechanism to CUMS. Both NGF (Figure 2(d)) and FGF-2 (Figure 2(e)) were downregulated by CUMS, while the swimming protocol also had no effect on their expression. Hippocampal generation of VEGF (Figure 2(f)) and IGF-1 (Figure 2(g)) was not influenced by chronic stress, but exercise significantly increased the expression of IGF-1 in non-CUMS rats. In parallel to the expression of BDNF, the mRNA levels of its downstream neurotrophic peptides NPY (Figure 2(h)) and VGF (Figure 2(i)) were synchronously inhibited by stress and restored by exercise. Chronic stress also downregulated Bcl-xl mRNA status (Figure 3(a)) and upregulated the expression of Bax (Figure 3(b)). The stress-induced decreased ratio of Bcl-xl/Bax indicated that the neural cells were shifted toward cell death (Figure 3(c)). However, exercise was found to promote Bcl-xl expression and increase the Bcl-xl/Bax ratio in non-CUMS rats. Meanwhile, in Stressed + Exercised group, the swimming protocol partly normalized the stress-induced imbalance between Bcl-xl and Bax.

## 4. Discussion

Neural atrophy has been reported in rodent chronic stress models and clinical postmortem studies of depressed patients. These findings indicate that depression can be considered as mild neurodegenerative disorder, and the increased neural cell loss may contribute to the pathogenesis of depression [22]. Recent studies have strengthened the role of the abnormalities in neurotrophic pathways in the psychopathophysiology of depression. It has been repeatedly documented that chronic stress and depression are associated with the abnormal neurotrophic signaling in the brain [23]. Exercise is an effective strategy for depression treatment. A key mechanism mediating the antidepressant action of exercise is induction of central and periphery growth factor cascades. Several classes of neurotrophic factors are implicated in the neural atrophy of depression and beneficial effects of exercise. Although most work has focused on BDNF, other growth factors also play a role in the stress-induced neural cell loss.

Several lines of evidence from animal and human researches converge on the idea that BDNF is essential for hippocampal synaptic plasticity and modulation of depression. The exercise-evoked BDNF release has been repeatedly postulated to underlie the antidepressant effects of physical activity [6, 23, 24]. In the present study, we confirmed the antidepressant effects of the swimming protocol, and, as previously reported, the induction of BDNF expression was also found in this study, suggesting the involvement of BDNF in the amelioration of depression by swimming exercise. Interestingly, in parallel to BDNF, a synchronous rise of NPY and VGF was also found in the Exercised group. The mRNA levels of NPY and VGF were inhibited in Stressed group, but were normalized in the Stressed + Exercised group. Both NPY and VGF are related to neuronal synaptic remodeling and could be highly induced by BDNF in vitro and in vivo [9, 25]. As NPY and VGF also actively participate in the neurotrophic hypothesis of depression and could be regulated by BDNF, our data argue that both BDNF and its downstream neurotrophic peptides, NPY and VGF, may play a role in the antidepressant effects of exercise.

Additionally, the effects of swimming exercise and stress exposure on other neurotrophic factors including GDNF, NT-3, NGF, and FGF-2 were also evaluated in this study. The exercise protocol had no influence on the four factors expression, but a significant reduction of NGF and FGF-2 was found in the Stressed group. While inhibiting NGF and FGF-2 expression, it has been reported that exposure to chronic stress upregulated NT-3 and GDNF [26, 27]. However, the results were inconsistent [28, 29]. In our study, we confirmed the increase of NT-3 and GDNF, which suggests a compensatory adaptive process to the stress-induced alterations of neurotrophic signaling in the hippocampus.

VEGF and IGF-1 are other two principle growth factors known to mediate the effects of exercise on the brain. Unlike the neurotrophins above, although both VEGF and IGF-1 can be synthesized in the brain, peripheral production is the main source of circulating VEGF and IGF-1. Both VEGF and IGF-1 are essential for exercise stimulated neurogenesis, and blocking either VEGF or IGF-1 signaling prevents exercise-induced neurogenesis in the hippocampus [30, 31]. However, the evidence of the influence of exercise on central generation of VEGF and IGF-1 is limited. In our study, we found that exercise only increased the hippocampal IGF-1 expression without any alterations of VEGF. Nevertheless, the significant change was not found between Stressed and Stressed + Exercised group, implying that the hippocampal production of IGF-1 does not play a key role in the antidepressant effects of swimming exercise. Since both VEGF and IGF-1 are increased in the periphery by exercise and can cross the blood-brain-barrier to enter the brain [32, 33], our data indicate that the beneficial effects of exercise appear to be mediated by the periphery genesis of the two factors, but not through the influence on paracrine effects of VEGF and IGF-1 in the hippocampus.

The neural apoptosis process is programmed and controlled by the cellular balance between antiapoptotic Bcl-xl and proapoptotic Bax expression. As previously reported, chronic stress downregulated the expression of Bcl-xl and upregulated Bax status [34, 35]. The altered balance indicates that the neural cell is shifted toward apoptosis. The beneficial effect of swimming exercise on neural survival was further validated by the restored balance between Bcl-xl and Bax. The enhanced expression of Bcl-xl was also found in the non-CUMS rats after the exercise protocol, suggesting that swimming exercise can not only reverse the apoptotic effect of stress but also improve the basal neural cell survival.

## 5. Conclusions

Collectively, since the neurotrophic signaling can regulate neural survival and Bcl-xl/Bax expression, and the reversed effects of swimming exercise on the stressinduced neurotrophic alterations were only found in BDNF and its downstream peptides (NPY and VGF) expression, our data suggests that the neurotrophic benefits of swimming exercise in the hippocampus are mainly through the BDNF signaling pathway, and NPY and VGF also synergetically play a role in the antidepressant action of swimming exercise.

## Figures and Tables

**Table 1:** Primer sequences used for the qPCR analysis.

Gene (accession no.)	Sense primer (5′-3′)	Antisense primer (5′-3′)	Amplicon length
BDNF (NM001270630)	TACCTGGATGCCGCAAACAT	GTAGAAATATTGCTTCAGTTGG	200 bp
GDNF (NM019139)	CAGAGGGAAAGGTCGCAGAG	CGTAGCCCAAACCCAAGTCA	95 bp
NT-3 (NM001270868)	GGGAGAGATCAAAACCGGC	TTGCGACGTTTTGCACT	136 bp
NGF (NM001277055)	ACATCAAGGGCAAGGAGG	GTGAGTCGTGGTGCAGTATG	164 bp
FGF-2 (NM019305)	TCCATCAAGGGAGTGTGTGC	TCCGTGACCGGTAAGTGTTG	139 bp
VEGF (NM001110333)	TATGTTTGACTGCTGTGGACTTGA	CAGGGATGGGTTTGTCGTGT	204 bp
IGF-1 (NM001082477)	TCAGTTCGTGTGTGGACCAG	CACAGCTCCGGAAGCAAC	117 bp
NPY (NM012614)	TGTTTGGGCATTCTGGCTGAGG	TTCTGGGGGCGTTTTCTGTGCT	205 bp
VGF (NM030997)	GATGAGTTGCCGGACTGG	CAACGCGTGATGGAAGTGAC	159 bp
Bcl-xl (NM001033670)	TGTGGCTGGTGTAGTTCTGC	CAGAAAAGCATTCCCGAGAG	402 bp
Bax (NM017059)	CTGCAGAGGATGATTGCTGA	GATCAGCTCGGGCACTTTAG	174 bp
*β*-Actin (NM031144)	CATCCTGCGTCTGGACCTGG	TAATGTCACGCACGATTTCC	116 bp
